# The detection and management of attempted fraud during an online randomised trial

**DOI:** 10.1186/s13063-023-07517-4

**Published:** 2023-08-04

**Authors:** Thomas A. Willis, Alexandra Wright-Hughes, Clare Skinner, Amanda J. Farrin, Suzanne Hartley, Rebecca Walwyn, Ana Weller, Mohamed Althaf, Stephanie Wilson, Chris P. Gale, Robbie Foy

**Affiliations:** 1 Leeds Institute of Health Sciences, University of Leeds, Worsley Building, Leeds, LS2 9JT UK; 2 Leeds Institute of Clinical Trials Research, University of Leeds, Leeds, UK; 3 Research Governance, Faculty of Medicine and Health, University of Leeds, Leeds, UK; 4 Centre for Human-Computer Interaction Design, City, University of London, London, UK; 5 Leeds Institute for Data Analytics, School of Medicine, University of Leeds, Leeds, UK; 6 Leeds Institute of Cardiovascular and Metabolic Medicine, University of Leeds, Leeds, UK; 7 Department of Cardiology, Leeds Teaching Hospitals NHS Trust, Leeds, UK

**Keywords:** Audit and feedback, Randomised fractional factorial experiment, Behaviour change, Trial misconduct, Online trial

## Abstract

**Background:**

Online studies offer an efficient method of recruiting participants and collecting data. Whilst delivering an online randomised trial, we detected unusual recruitment activity. We describe our approach to detecting and managing suspected fraud and share lessons for researchers.

**Methods:**

Our trial investigated the single and combined effects of different ways of presenting clinical audit and feedback. Clinicians and managers who received feedback from one of five United Kingdom national clinical audit programmes were emailed invitations that contained a link to the trial website. After providing consent and selecting their relevant audit, participants were randomised automatically to different feedback versions. Immediately after viewing their assigned feedback, participants completed a questionnaire and could request a financial voucher by entering an email address. Email addresses were not linked to trial data to preserve participant anonymity. We actively monitored participant numbers, questionnaire completions, and voucher claims.

**Results:**

Following a rapid increase in trial participation, we identified 268 new voucher claims from three email addresses that we had reason to believe were linked. Further scrutiny revealed duplicate trial completions and voucher requests from 24 email addresses. We immediately suspended the trial, improved security measures, and went on to successfully complete the study.

We found a peak in questionnaires completed in less than 20 seconds during a likely contamination period. Given that study and personal data were not linked, we could not directly identify the trial data from the 268 duplicate entries within the 603 randomisations occurring during the same period. We therefore excluded all 603 randomisations from the primary analysis, which was consequently based on 638 randomisations. A sensitivity analysis, including all 961 randomisations over the entire study except for questionnaire completions of less than 20 seconds, found only minor differences from the primary analysis.

**Conclusion:**

Online studies offering incentives for participation are at risk of attempted fraud. Systematic monitoring and analysis can help detect such activity. Measures to protect study integrity include linking participant identifiers to study data, balancing study security and ease of participation, and safeguarding the allocation of participant incentives.

**Trial registration:**

International Standard Randomised Controlled Trial Number: ISRCTN41584028. Registration date is August 17, 2017.

## Background

Online studies offer an efficient method of recruiting participants and collecting data. They are increasingly being used in health research, including for randomised trials. A PubMed search for randomised controlled trials featuring ‘online’ or ‘internet’ in the title found a total of 2742 records, with steady growth in the annual rate of such publications from the first in 1989 to 269 on 20 December 2022. Online trials can be designed so that they can filter potential participants for eligibility, consent and randomise participants, deliver interventions, collect data, and thank participants.

We completed an online trial which involved all of these steps as part of a multiphase optimization strategy (MOST), a methodological approach for building, optimising, and evaluating multicomponent interventions [1]. MOST comprises three steps: preparation, laying the groundwork for optimisation by conceptualising and piloting components; optimisation, conducting trials to identify the most promising single or combined intervention components; and evaluation, a definitive randomised trial to assess intervention effectiveness.

Our online trial corresponded to the second phase of MOST. We investigated the single and combined effects of different components of clinical audit and feedback. Audit and feedback is commonly used in healthcare quality improvement [2]. It aims to improve the uptake of recommended practice by reviewing clinical performance against explicit standards and directing action towards areas not meeting those standards [3]. There are many potential ways of delivering feedback (e.g. varying comparators, display characteristics), and we investigated the single and combined effects of six such components. We randomised clinical and managerial staff who received feedback from national clinical audit programmes to different versions of feedback and assessed effects on self-reported intended enactment of clinical recommendations [4].

However, the delivery of our online trial was disrupted by potentially fraudulent activity. In this paper, we describe the rationale for our original approach to recruitment, and how this inadvertently left our trial exposed to attempted fraud by one or more participants. We outline how we identified the incident and our remedial action to ensure trial integrity.

## Methods

### Overview of design

We conducted an online fractional factorial trial, described in full elsewhere [4]. Six modifications to feedback were each operationalised in two versions (ON with the modification applied, OFF without modification) and applied within audit report excerpts for five different national clinical audits. For example, in one modification (‘multimodal feedback’), the performance result was provided in text format and accompanied by a graphical display of performance data (ON) versus text format alone (OFF). Participants were randomised to receive one of 32 combinations of the modifications, stratified by national audit programme. We informed participants that the excerpt contained simulated but realistic data. After viewing the excerpt, participants were asked to complete a short questionnaire. The primary outcome was intended enactment of audit standards.

### Setting and participant recruitment

We developed our online trial in partnership with five United Kingdom (UK) national clinical audit programmes: the Myocardial Ischaemia National Audit Project (MINAP), the National Comparative Audit of Blood Transfusion (NCABT), the National Diabetes Core Audit (NDA), the Paediatric Intensive Care Network (PICANet), and the Trauma Audit and Research Network (TARN). Each audit programme emailed study invitations to their own networks of clinicians, managers, and administrators, all of whom were eligible to participate. These networks were mainly hospital-based, with the exception of the National Diabetes Core Audit, which included most general practices in England and Wales.

The email invitations included a link to the trial website, through which recipients could consent to participate, select their relevant audit, and provide information about their role and organisation. Participants were randomised automatically, stratified by audit, to one of the 32 different fictional versions of performance feedback. Immediately after viewing their assigned feedback, participants were required to complete a 14-item questionnaire assessing their responses to the feedback, mostly using 1–5 Likert scales with radio buttons. We aimed to maximise ease of completion. Participants could then request a £25 voucher by entering an email address that was manually checked by an administrative member of staff before the voucher was sent. We deliberately placed the request for email addresses at the end of the survey, after participants had entered, in line with the usual practice for survey design in placing requests for personal data after the main survey questions. To preserve participant anonymity, email addresses provided for voucher requests were not linked to trial data. A ‘dashboard’ allowed real-time monitoring of the number of participants entering the study and completing the questionnaire for each audit programme and the total number of vouchers claimed; it did not include any automated data validity checks.

We based our recruitment approach on several assumptions. First, financial incentives may improve study recruitment [5], and we judged that £25 reasonably recognised time participating by busy health professionals, which we estimated as around 20 minutes. Second, we trusted a manual rather than automatic process to check voucher claims and distribute vouchers. Third, we recruited participants via national audit networks but were aware that the invitation could be shared more widely beyond the original target population. We therefore set predefined recruitment limits for each of the five audit programmes. Fourth, we made the critical decision to ensure anonymity of study data by not retaining a link between personal data and study responses. We considered that anonymity would also enable more honest responses, reduce social desirability bias, and simplify arrangements for protecting personal data. In addition, the university responsible for hosting the website advised minimising retention of personal identifiable data. We positioned the only request for personal identifiable data, name, and email address, after questionnaire completion for participants to claim vouchers.

## Results

### Detection of unusual study activity

**Fig. 1:**
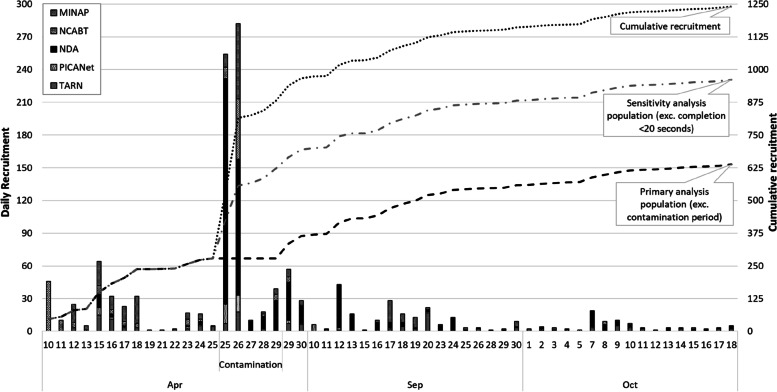
Pattern of recruitment to the online trial

The study launched 10 April 2019 when the five national clinical audits emailed their initial distribution lists, totalling around 2000 recipients. We reached half of our target of 500 randomised participants within a fortnight. The NDA’s distribution list was far larger than that of the other four audits, and so we had initially limited the number of invitations to approximately 500 general practices. However, given a relatively low response for this audit, we extended the invitation to all 7300 practices on the NDA distribution list. This invitation was sent 25 April 2019. We then observed a sharp increase in responses, prior to and over one weekend (Fig. 1).

We completed a pre-planned, secure download of voucher request data on the following Monday morning, 29 April 2019. Our study administrator identified 268 new voucher claims from three email addresses. We were subsequently able to link these email addresses to one general practice, using a combination of a telephone number that had been logged as calling our study enquiry number, and the name provided in the relevant email addresses. The caller had enquired as to whether participants could complete the study more than once.

We then inspected the study data and found emerging unusual patterns compared to earlier validity checks. We consulted our independent study steering committee, as well as the study sponsor, and suspended the online trial to allow further investigation.

### Impact on study data

Closer scrutiny of voucher request data, which included the submitted email address and name (where available), revealed that duplicate completions and voucher requests were associated with 24 email addresses (Table 1). This consisted of 17 email addresses submitted for two voucher requests each and a further four email addresses with between three and five requests each. A total of 268 voucher requests originated from three email addresses.

**Table 1:** Number of voucher requests per email address during the initial recruitment period

Number of voucher requests per email address	*N* (%)
**1**	514 (95.5%)
**2**	17 (3.2%)
**3**	2 (0.4%)
**4**	1 (0.2%)
**5**	1 (0.2%)
**22**	1 (0.2%)
**77**	1 (0.2%)
**169**	1 (0.2%)
**Total**	538 (100%)

These three separate addresses all shared a common name, suggesting that they were linked to one individual. This individual’s experiment activity occurred over 4 days immediately following the NDA invitation to a wider list of general practices. Indeed, the bulk of responses during the suspect period related to this audit (Table 2); however, we also observed unusual patterns in the data for the other four audits.

**Table 2:** Number of randomisations across audits and time spent on trial components by contamination period

	**Randomisations during the contamination period:**		
	**Yes**	**No**	**Total**
**Total randomisations—*N***	603	638	1241
**Audit**			
MINAP	89 (14.8%)	178 (27.9%)	267 (21.5%)
NCABT	73 (12.1%)	102 (16.0%)	175 (14.1%)
NDA	380 (63.0%)	204 (32.0%)	584 (47.1%)
PICANet	33 (5.5%)	36 (5.6%)	69 (5.6%)
TARN	28 (4.6%)	118 (18.5%)	146 (11.8%)
**Time on audit report (seconds)**			
*N***—**total viewed audit report to end	597	629	1226^1^
Median (range)	13.5 (0.5, 14,302.5)	66.5 (0.5, 70,512.0)	43.5 (0.5, 70,512.0)
Interquartile range	(2.0, 60.5)	(31.0, 136.0)	(7.0, 98.5)
**Time on questionnaire (seconds)**			
*N***—**total completing questionnaire	547	566	1113
Median (range)	31.0 (3.5, 19,783.0)	159.0 (2.5, 16,320.0)	113.5 (2.5, 19,783.0)
Interquartile range	(13.0, 139.0)	(97.5, 255.5)	(19.0, 205.0)
** > 20 seconds on questionnaire**			
Yes	288 (52.7%)	545 (96.3%)	833 (74.8%)^2^
No	259 (47.3%)	21 (3.7%)	280 (25.2%)
NA**—**did not complete questionnaire	56	72	128^1,2^

*MINAP *Myocardial Ischaemia National Audit Project, *NCABT* National comparative audit of blood transfusion, *NDA* National Diabetes Audit, *PICANet* Paediatric Intensive Care Network, *TARN* Trauma Audit Research Network

^1^* N* = 15 randomisations did not view the audit report to the end, and a further *N* = 113 randomisations viewed the audit report to the end but did not complete the subsequent questionnaire, resulting in a total *N* = 128 randomisations without a completed response to the questionnaire

^2^ *N* = 961 included in secondary analysis population (all randomisations minus those completing the questionnaire in < 20 seconds), sensitivity analysis using this population was conducted on complete data, thus comprising *N* = 833 randomisations with completed questionnaires

We were able to identify a likely contamination period. This was defined based on the date and timing of the second NDA invitation (2:50 pm, 25 April 2019) and identification of the duplicate activity from the voucher requests extracted from the experiment (11:35am, 29 April 2019). Between identification of the duplicate activity and temporary suspension of the experiment, 2 days later, we found no further duplicates.

We found that several questionnaires concerning the four hospital-based audits (MINAP, NCABT, PICANET, TARN) had been completed during the likely contamination period by respondents who identified their work setting as primary care. This was surprising: there was no clear reason why primary care professionals would choose to review audit data concerning topics such as paediatric intensive care or blood transfusions. We contacted the three email addresses that had accounted for 268 voucher requests and the replies confirmed that the experiment had been completed for all five clinical audits.

**Fig. 2:**
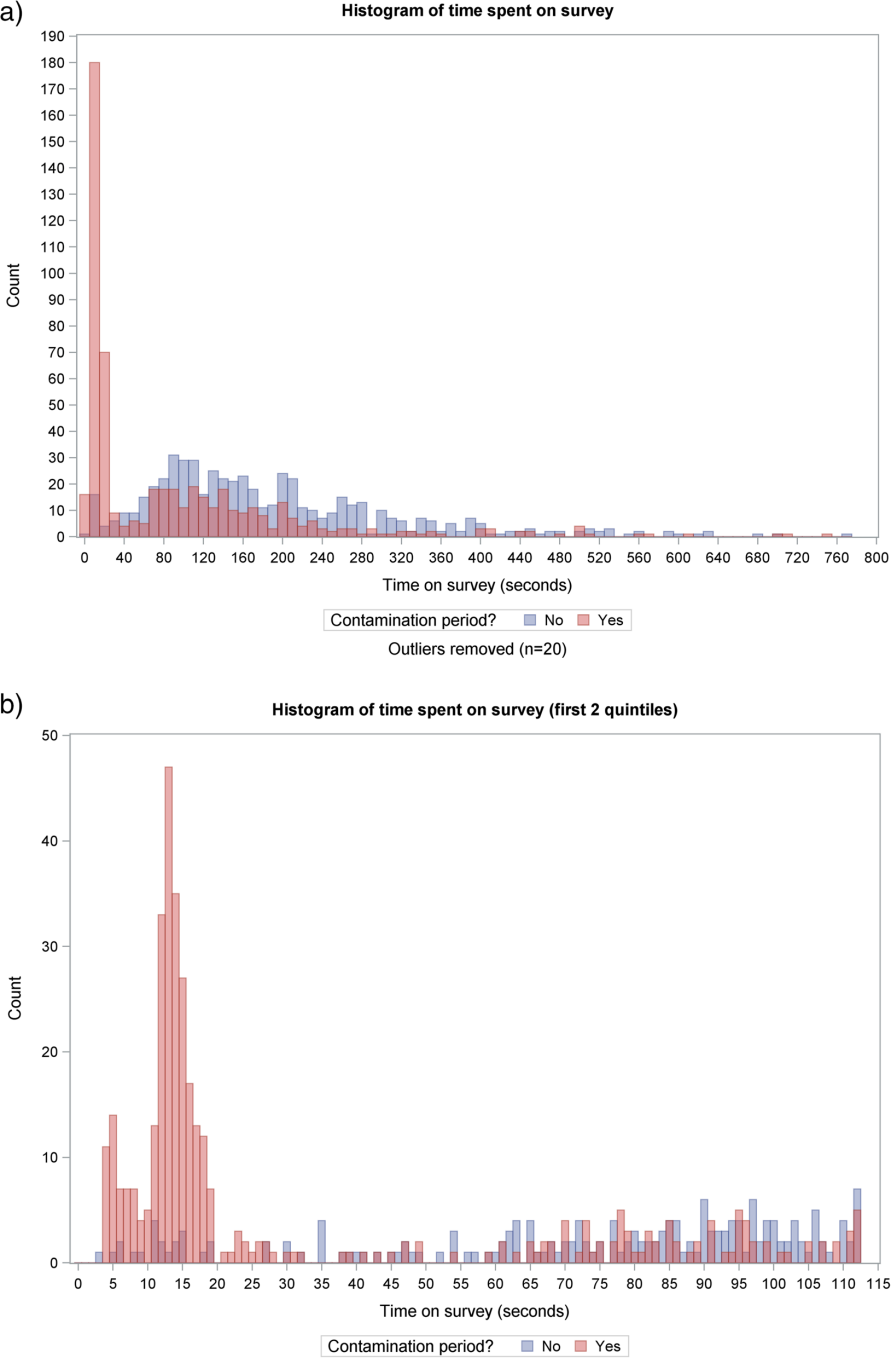
Histogram of time spent on questionnaire

We compared trial data collected before, during, and after the likely contamination period. Figure 2 presents the distribution of time spent completing the questionnaire and shows a peak in questionnaires completed in less than 20 seconds during the contamination period. Such a short completion time seemed highly unlikely to represent considered engagement with a 14-item questionnaire. Of those completing the questionnaire, only 3.7% spent less than 20 seconds completing it outside of the contamination period; this increased to 47.3% during the contamination period (Table 2).

Comparison of the two periods revealed other patterns. For example, a reduction in the time spent viewing the audit report was apparent. Furthermore, a greater proportion of respondents had selected ‘completely agree’ to all questionnaire items, suggesting that they had clicked quickly through the questionnaire with minimal engagement. However, it was plausible that both the time spent on the audit excerpt and the questionnaire response pattern could be associated with the version of the audit that participants were randomised to receive: some versions included minimal content, which might therefore require less time to interpret. Consequently, we did not use these indicators to identify suspect responses.

### Primary and secondary analysis populations

Given that study and personal data were not linked, we were unable to directly identify the study data from the 268 duplicate entries from the total 603 randomisations which took place during the same period. We therefore produced two datasets for analysis, aiming to protect trial validity by using objective criteria to include only independent, non-suspect responses. The primary analysis population comprised all randomisations and questionnaire responses received outside of the defined contamination period; this included 638 randomisations and 566 completed questionnaires and excluded the 603 randomisations and 597 responses within the contamination period. The secondary analysis population included all randomisations excluding the 208 with questionnaire responses completed in less than 20 seconds; this included 961 randomisations and 833 completions.

### Action taken

Upon discovery of the suspicious activity, we promptly reported the incident to our independent study steering Committee, the research funder (National Institute for Health and Care Research), the University of Leeds School of Medicine Research Ethics Committee that had approved the study, and the study sponsor. We submitted a report to NHS Counter Fraud and then to the Department of Health and Social Care as the ultimate study funder. We informed our Patient and Public Involvement panel, which expressed concern about the misuse of public funding.

We revised the study recruitment materials (email invitation and study landing page) to emphasise that participants should complete the study once only. We enhanced study security by adding a screening page prior to study entry. This required participants to submit an NHS or Health and Social Care (Northern Ireland; HSCNI) email address before proceeding. The submitted email address was then subjected to two automated checks: first, whether it had been submitted previously, and second, whether it was a plausible NHS or HSCNI email address. Anyone attempting to use an address rejected by either check was unable to proceed.

We subsequently relaunched the online trial for a pre-specified period (5 September 2019 to 18 October 2019). By the end of the second recruitment period, there were a total 1241 randomisations and 1113 experiment completions, including a total 1080 voucher requests. During the second recruitment period, we identified two experiment completions associated with two similar NHS email addresses (Table 1), despite the additional security measures. We also identified a further 16 experiment completions during the first recruitment period and then again using similar NHS email addresses when the trial re-opened. It is uncertain whether these were intentional duplications or NHS email addresses used by more than one individual with similar identifiers (e.g. surname).

We shared a written summary of our evidence with the general practice that we had linked to the unusual trial activity. We received a response from the senior partner expressing concern that they had been affected by ‘cybercrime’. We did not send any vouchers to the duplicated email addresses.

### Study completion

We successfully completed our trial after the second recruitment period. We identified the effects of varying the content and format of feedback from national clinical audits on health professionals’ responses [4]. Our initial concerns that requesting email addresses at study entry might deter participants were unfounded: there was minimal difference in response activity between the first and second recruitment periods. Our sensitivity analysis, which excluded participants who spent less than 20 seconds completing the questionnaire and therefore retained data from the contamination period that we considered to be less risky, provided a larger sample size with greater power and found only minor differences from the primary analysis.

## Discussion

Online studies offer the potential for efficient and practical recruitment of large numbers of participants within a relatively brief period. However, our experience illustrates that this is not always necessarily a good thing. Without appropriate monitoring and safeguards, online studies are potentially vulnerable to abuse and ‘careless’ responses, each of which undermines study validity [6–9]. Whilst we cannot claim to be the first study team to experience attempted fraud affecting an online experiment, it is almost certain that we will not be the last. Therefore, we were motivated to write a transparent account to remind others of risks involved in delivering an online trial and consider measures to protect study integrity.

Three study design features contributed to the exploitation of our study and consequent wastage of research data. First, financial incentives may improve response rates [5], yet may be associated with duplicate responses [6]. We reflected on whether our incentive was too large, or even necessary. The fact that we only modestly exceeded our recruitment target, however, suggests the level of incentive was appropriate for our study population. Second, we had trusted that all participants would readily recognise which national audit programmes were relevant to them, e.g. it would be patently obvious to a primary care professional that a paediatric intensive care or blood transfusion audit would not be relevant to their clinical roles. This transpired to be a false assumption, at least when we encountered one or more potential participants who appeared to be mainly interested in maximising personal financial reward. Third, we applied an overly cautious interpretation of data protection principles. All UK research activity that involves the processing of personal data must be conducted in accordance with the Data Protection Act [10]. This describes how personal data (i.e. information about living people who can be identified from that data) should be handled. It includes requirements that data be kept safe and secure, processed fairly and lawfully, and that data collected are relevant and limited to what is necessary. If personal data are only collected for administrative purposes (e.g. contact details, as was the case with our trial), they should be kept separate from research data [11]. Institutions and researchers, rightly, take these responsibilities seriously; there are legal, financial, and reputational risks if personal data are mishandled. It is tempting for researchers and institutions to default to segregating personal and study data to avoid such risks, as we did. In hindsight, data concerning how health professionals respond to fictional variations of feedback is not particularly sensitive. It would have been reasonable to keep these data linked via a pseudonymised key, especially given the offer of financial incentives. Retaining the ability to link data in order to identify duplicate entries would also be acceptable within the principles of the UK General Data Protection Regulation (GDPR), as outlined by the UK Information Commissioner’s Office [12]. Linking the identifiable data to the research data would not have prevented what occurred, but it would have enabled us to clean the data by removing the relevant entries.

We offer some suggestions for other researchers planning online studies based on our experience and that of others (Table 3) [6–9, 13].

**Table 3:** Suggestions to protect the integrity of online research

Consider what is essential to meet ethical safeguards and data protection regulatory requirements. Is there a strong reason to remove linkage of personal and (non-sensitive) study data?
Assess the balance between study security and ease of participation. Requesting limited personal information, such as email addresses, at study entry may not reduce response rates
For surveys to specific professional groups, include a requirement, where possible, for associated email addresses. For example, as here, healthcare professionals would be required to provide health system-based emails. Whatever the participant group, ensure that checks against duplication are included from the design stage and, where possible, are automated
Attempt to visualise problematic scenarios. A single individual can exploit an existing vulnerability. What are the vulnerabilities of your study and how might they be exploited? If a problem arose, would it be possible to identify and exclude suspect data with certainty?
Regularly monitor aspects of collected data, ideally using a live dashboard. Consider unusual emerging patterns or trends; simple checking of recruitment totals may mask problems until it is too late
Examine study data to look for expected and unexpected anomalies. Consider whether procedures for identifying suspect or duplicate responses can be fully automated or if manual checks are required and schedule appropriately
Unless there is high confidence in study security, use manual rather than automated delivery of incentives
Ensure that at least one person can access study systems and extract detailed monitoring data. Rapid responses can be critical in damage limitation

## Conclusion

Online studies, and particularly those offering incentives for participation, are at risk of encountering fraudulent activity. Whilst live monitoring and systematic analysis of data can help detect such activity, researchers can build in measures during the design stage to help protect study integrity. These may include achieving a balance between study security and ease of participation and maintaining safeguards in the allocation of any participant incentives.

## Data Availability

The datasets generated and analysed during the current study are available from the corresponding author on reasonable request.

## Abbreviations

GDPR: General Data Protection Regulation
HSCNI: Health and Social Care Northern Ireland
MINAP: Myocardial Ischaemia National Audit Project
NCABT: National comparative audit of blood transfusion
NDA: National Diabetes Audit
NHS: National Health Service
PICANet: Paediatric Intensive Care Network
TARN: Trauma Audit Research Network
UK: United Kingdom
